# Surgical Cases and Observations

**Published:** 1845-05

**Authors:** James Syme

**Affiliations:** Professor of Clinical Surgery in the University of Edinburgh, and Surgeon to the Queen in Scotland


*Surgical Cases and Observations. By* James Syme, Esq., *Professor of Clinical Surgery in the University of Edinburgh, and Surgeon to the Queen in Scotland.*

Case 1. *Dislocation of the Shoulder-joint, of seven weeks standing; Reduction.*—Euphemia Steele, aged 55, was admitted on the 25th of December last, with dislocation of the left shoulder-joint. It appeared, that on the 5th of November, she had fallen upon her side, and in consequence suffered much pain from of the left arm;—that she had been under surgical treatment from the 13th to the 27th, for a bruise on the elbow;—and that at the latter date, as there was nothing perceptibly wrong with the elbow, her continued complaint of pain from the shoulder downwards was discredited, while a flattening noticed under the acromion, was attributed to emaciation of the limb, from the want of exercise. A month having afterwards elapsed without any improvement, the patient applied at the Minto House Dispensary, and being there found to labour under dislocation of the shoulder, was placed under my care in the hospital.

On the 26th, after immersion in the warm bath for an hour, reduction was attempted by extending the arm above the elbow in a line with the trunk—but though the head of the bone, which lay forwards on the inner side of the coracoid process, was made to move considerably towards the socket—it could not be fairly replaced. Two days afterwards, another attempt proved more effectual. I, this time, as before, laid the patient upon her back on a table—secured a hair cushion in the axilla by means of a stuffed leather belt, fastened to a ring in the wall, and then extended by pulleys acting upon a skein of worsted attached by the "clove hitch" to the wrist, instead of the arm above the elbow. My reason for this alteration was, that during the former trial I had remarked the integuments of the arm and shoulder to be extremely tense, and hence concluded that the force might be more efficient if it were to act upon a more distant part of the limb. In accordance with this expectation it was immediately perceived that the bone yielded much more readily than it had done on the previous day; and without any snap being heard or felt, the patient soon exclaimed that her shoulder was right. On examination it was found to be so, though the slightest movement of the arm, in the way of abduction, caused the bone to quit its place, into which, however, it could be easily returned again, by slight pressure of the fingers in the axilla. A bandage applied so as to confine the elbow close to the side, kept the joint secure until its natural connexions were sufficiently restored to prevent any risk of displacement, and the patient was discharged on the 18th January.

The means employed for reducing dislocation of the shoulder-joint, should be varied according to circumstances, especially the period of its duration. Within a few hours after the injury, I have repeatedly effected reduction without any assistance, by placing one hand on the acromion, and then, having bent the fore-arm to a right angle, suddenly drawing the elbow backwards, so as at the same time to rotate the hand outwards.

The effect of this movement is well illustrated by a case which lately fell under my notice. The patient came from the country, a distance of twelve miles, for the purpose of having a dislocation of his shoulder reduced. Seeing from the position and powerless appearance of the arm, that the bone was displaced; and having felt, by putting my arm under his clothes, that its head lay in the axilla, I desired him to take off his coat. No sooner had this, with some assistance, been accomplished, than he declared that he felt his shoulder quite right, which it really was, no doubt from the action required for withdrawing his arm from the sleeve.

In ordinary cases, of from five hours to as many days duration, the most convenient method is to seat the patient upon a chair, and pull the arm at a right angle with the chest. If the resistance cannot be readily overcome in this way, it may be warrantable to employ the rude, but powerful means of extending by the hand against the heel in the axilla. In a dislocation of two weeks standing, which had been previously subjected, without success, to several very forcible attempts, I accomplished reduction almost instantaneously, by desiring one of my pupils to place his foot in the patient's axilla, and pull his hand. When, from the lapse of time, a still greater degree of difficulty is to be anticipated, the assistance of pulleys becomes proper, together with the use of some means to lessen the force of muscular contraction. For this purpose, tartar emetic, bleeding, and the warm bath, are generally employed. In the earlier part of my practice, I generally combined the effects of these nauseating and depressing influences; but the one last mentioned seems to be quite sufficient of itself; and being not unpleasant to the patient, either at the time or afterwards, the others had better be omitted. From the variety of directions which have been adopted by different practitioners, for extending the arm, it might seem as if the degree of force were of more consequence than the line of its operation; while the truth, I believe, is, that success depends very much upon the limb being held, during the extension, near the side of the body, so as to relax the pectoral and dorsal muscles, which constitute the margin of the axillary hollow. Hence the success attending the plan of pulling against the heel in the axilla; and hence the propriety of extending in a line with the trunk, when the amount of difficulty anticipated suggests the sacrifice of convenience for efficiency. The extending power may act either on the arm above the elbow, or on the wrist. The former situation allows the fore-arm to be used as a lever for causing rotation, but exposes the integuments and muscles to compression, which must always be opposed to the object in view,—and as in the case above related, may prevent it from being attained. That it does not necessarily thus impede success, however, will appear from the following cases.

Case 2. *Dislocation of the Shoulder-joint, of seven weeks standing, reduced.*—William Stewart, aged 56, was admitted on the 3d December, 1840, seven weeks after sustaining a dislocation of the right shoulder-joint, for which he had been treated in the country by a bone-doctor, as suffering merely from the bruise occasioned by a fall on his side. After having been an hour in the bath, he was laid horizontally, and subjected to extension by means of the pulleys, in the direction of the long axis of the body. The bone regained its place without any snap, but escaped on the extension being discontinued,—and therefore, when again reduced, was secured by a bandage confining the arm to the side.

Case 3. *Dislocation of the Shoulder-joint, of four weeks standing: Reduction.*—James Grieve, aged 50, was admitted on the 2d January, 1844, four weeks after having his left shoulder-joint dislocated. He had been under the care of a surgeon, who had tried to reduce the bone, and assured him he had done so, though no relief or alteration in his feelings was afforded. He was immediately subjected to the pulleys—without success; but next day, after being an hour in the bath, had the bone restored to its place.

CASE 4. *Dislocation of the Shoulder-joint, of four weeks standing: Reduction.*—Elizabeth Gair, aged 53, was admitted on the 30th of November, 1844. She stated, that on the fourth of the same month she had fallen on her right elbow; that a surgeon to whom she immediately afterwards applied, told her the shoulder-joint was dislocated, and tried to reduce it by means of his heel in the axilla, and that then (experiencing no relief, though assured the bone was replaced,) she had recourse to a "*bone-setter*," who made an attempt which did not prove more successful. On the 2d of December, after being in the warm bath, the patient was laid upon a table, with a hair cushion secured in her axilla. Extension was then made from above the elbow by pulleys, and the bone very soon returned into its socket, with a distinct snap. As abduction of the arm was found to cause renewal of the displacement, I bandaged the limb as usual to the side.

In Dislocation of the Hip-Joint upwards and backwards, the extending force may act either upon the ankle or above the knee; but as rotation of the limb very considerably conduces to replacement, and as this movement is best effected by means of the leg used for a lever, while the knee is bent, the preference should be given to extension from above the knee. It may be added, that the extensors of the hip, being flexors of the knee, are put very much upon the stretch, if the knee is kept straight, while the limb is extended, as it ought to be, in the direction which the thigh bone assumes in this form of dislocation.

CASE 5. *Dislocation of the Hip-joint of five weeks standing:—Reduction.*—James Millar, aged 40, was admitted on the 23d of January last, five weeks after being overwhelmed by a fall of earth, while his legs were crossed. The left limb was nearly two inches shorter than the right one, and in all other respects exhibited very distinctly the characters of dislocation of the hip-joint on the dorsum ilii. Next day, after the usual preparation in the warm bath, he was subjected to extension from the ankle, without success, the lever afforded by the foot for causing rotation, being obviously very inefficient, and the muscles on the back part of the thigh feeling extremely tense. The pulleys were then made to act above the knee, and speedily restored the bone to its place.

CASE 6. *Dislocation of the Thigh-bone, upwards and backwards, primarily, on the dorsum ilii, secondarily, into the ischiatic notch:—Reduction.*—James Hunter, aged 18, was admitted on the 13th February last, suffering from the effects of an injury caused the preceding day by a railway carriage, which threw him down with great violence. In addition to some superficial bruises, the right thigh-bone was dislocated upwards and backwards. The process of reduction was conducted as usual, and apparently with success, as the bone distinctly moved, and grated under my hand, which rested upon the trochanter, the characteristic deformity at the same time disappearing. The injured limb, instead of being almost two inches shorter, and turned inwards, so that the toes rested upon the instep of the other foot, seemed hardly at all diminished in length, and had become quite straight in its direction. But the limb, though nearly, was not quite of the proper length; the foot, though no longer inverted, did not admit of rotation outwards; the thigh had a stiff constrained aspect; and the patient's back, instead of resting flat upon the mattrass, remained in an arched form, unless the thigh was raised into a position of semiflexion on the pelvis. These characters denoted dislocation of the thigh-bone into the ischiatic notch, and led to a repetition of the process for reduction, which very soon had the desired effect, and the appearance of the limb became in every respect natural.

The important feature of this case is the secondary dislocation that took place during the reduction. In more than one instance which has fallen under my observation, the same change of circumstances occasioned the serious error of supposing that the bone had returned to its proper place, while it had merely shifted into the notch; and as that excellent authority, Sir A. Cooper, though he has warned against the risk of this occurrence in reducing dislocation into the foramen ovale, has not noticed it with regard to the more common case of dislocation on the ilium, or pointed out the deceitful alteration of appearances so induced, I hope the instance here related will not be without use.

In regard to the comparative frequency of the different dislocations to which the hip-joint is liable, Sir A. Cooper has stated their respective numbers in twenty cases that had fallen under his own observation,—on the dorsum ilii, 12; into the ischiatic notch, 5; into the foramen ovale, 2; and on the pubis, 1. It is curious that of 10 cases which have been reduced in my practice in the Royal Infirmary during the last twelve years, the proportion is almost precisely the same.

*On the Pubis.*

Alexander Grieve, aged 23,—recent.

*Into the Foramen Ovale.*

Hamilton Hamill, aged 45,—recent.

*Into the Ischiatic Notch.*

James Inglis, aged 56,—recent.
Lawrence Smith, aged 18,—two weeks standing.

*On the Dorsum Ilii.*

William Scott, aged 36,—9 weeks standing.
Elizabeth Waters, aged 26,—6 weeks do.
William Thomson, aged 50,—recent.
Jane Millar, aged 40,—5 weeks standing.
James Hunter,—recent.
A boy, aged 14,—recent.

There was another dislocation into the ischiatic notch, which I did not reduce, as the patient was dying from rupture of the bladder.

*Lond. and Ed. Monthly Med. and Surg. Journ.*

